# Dermabrasion Using Surface Anesthesia for Post-Cleft Lip Surgery Scars

**DOI:** 10.7759/cureus.88329

**Published:** 2025-07-19

**Authors:** Teruyuki Niimi, Hiroo Furukawa, Ken Kitagawa, Chisato Sakuma, Nagato Natsume

**Affiliations:** 1 Division of Research and Treatment for Oral and Maxillofacial Congenital Anomalies, School of Dentistry, Aichi-Gakuin University, Nagoya, JPN; 2 Cleft Lip and Palate Center, Aichi Gakuin University Dental Hospital, Nagoya, JPN

**Keywords:** cleft lip surgery, dermabrasion, hydrocolloid dressing material, scar, surface anesthesia

## Abstract

Dermabrasion is a minimally invasive technique effective for improving postoperative scarring in cleft lip patients. While commonly performed under local anesthesia, needle-related pain often causes discomfort, particularly in children. To address this, we evaluated the efficacy of dermabrasion under surface anesthesia using lidocaine tape. A total of 92 patients underwent dermabrasion following the application of lidocaine tape for varying durations. Results showed that painless treatment was achieved in 25% of patients with 30-59 minutes of application, 61% with 60-89 minutes, and 67% with over 90 minutes. No patient required injectable anesthesia, and all cases showed favorable cosmetic outcomes without complications. Our findings suggest that applying lidocaine tape for at least 60 minutes significantly improves patient comfort and eliminates the need for injectable anesthesia. This approach is effective and less invasive for scar management after cleft lip surgery.

## Introduction

Dermabrasion is a mechanical skin resurfacing technique that has been utilized for over a century to treat a wide range of dermatologic conditions. First introduced in the early 20th century, the procedure involves controlled abrasion of the epidermis and upper dermis using a rapidly rotating instrument such as a wire brush, diamond fraise, or dental burr. By physically removing the superficial layers of the skin, dermabrasion stimulates re-epithelialization and collagen remodeling and ultimately leads to smoother skin texture and reduced scar prominence. Historically, dermabrasion has been used to treat acne scars, traumatic scars, surgical scars, and pigmentary disorders and remains a cost-effective and widely accessible alternative to more technologically advanced options such as laser resurfacing [1-4].

Clinical studies have demonstrated favorable outcomes with dermabrasion in both aesthetic and reconstructive contexts. Patient satisfaction rates for scar revision using dermabrasion have been reported to exceed 80% in several cohorts, with notable improvements in texture, pigmentation, and contour irregularities [5,6]. Despite its effectiveness, the procedure can be associated with significant intraoperative discomfort, necessitating the use of local injectable anesthesia. This is particularly problematic in pediatric populations, where needle-related pain and anxiety often result in poor compliance and negative procedural experiences [7].

In the field of cleft lip and palate surgery, dermabrasion serves a specialized role in refining postoperative scars on the lip and nasal base once primary healing has occurred. These scars, while functionally stable, may remain cosmetically conspicuous and socially distressing, especially for children and adolescents. At our institution, we have routinely employed dermabrasion to improve the appearance of these scars in patients with otherwise satisfactory surgical outcomes. However, the use of injectable anesthesia in this context presents challenges due to both psychological discomfort and the technical difficulty of re-anesthetizing previously operated tissue.

To address these concerns, we have implemented the use of surface anesthesia via lidocaine adhesive tape (Penles®, Nitto Denko Corporation, Osaka, Japan), which delivers a localized dose of 60 mg lidocaine without the need for needle injection. While topical anesthetics such as eutectic lidocaine-prilocaine creams have been utilized in minor dermatologic procedures, the literature remains sparse regarding their application in dermabrasion, particularly in pediatric and post-cleft repair contexts.

The purpose of this report is to evaluate the efficacy of lidocaine tape for pain control during dermabrasion in cleft lip patients and to determine the optimal application time needed to achieve sufficient analgesia. We retrospectively analyzed the outcomes of 92 patients who underwent dermabrasion with pre-procedural application of lidocaine tape, assessing the relationship between anesthetic application duration and the need for additional analgesia. Our findings suggest a practical, minimally invasive alternative to injectable anesthesia, particularly suited for scar management in children.

## Technical report

**Figure 1:**
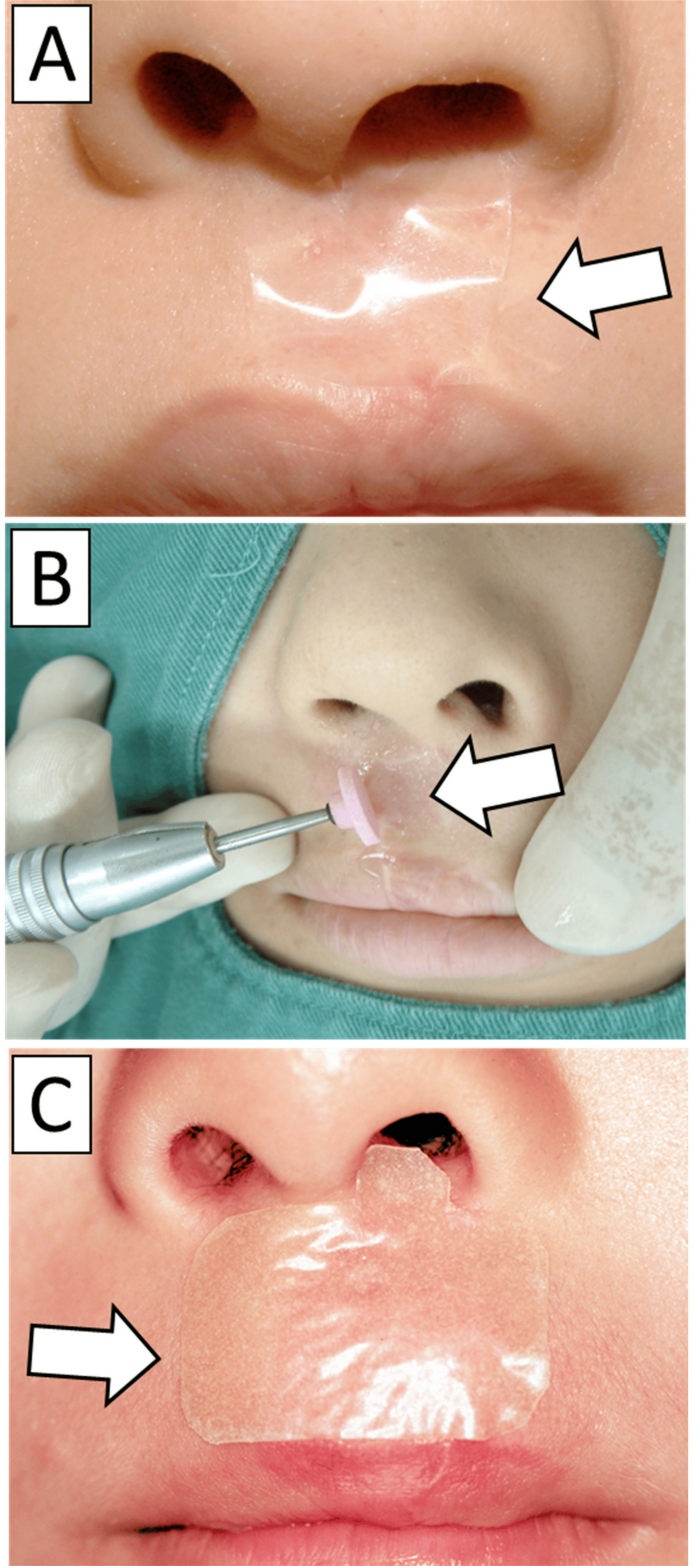
Procedure of dermabrasion A: Lidocaine tape is put on the treatment site (arrow). B: Scar is abraded using a dental stone wheel cooled by rinsing with normal saline (arrow). C: Hydrocolloid dressing is applied after dermabrasion (arrow).

Patients undergoing dermabrasion for scarring after cleft lip surgery have lidocaine tape (Penles® tape, Nitto Denko Corporation, Osaka, Japan) applied to the treatment site. After leaving it in place for at least 30 minutes, the scar tissue is peeled off using a dental plaster wheel cooled with saline solution. If pain occurs during dermabrasion, apply a sponge soaked in 4% lidocaine. Additional anesthesia was administered at the slightest sign of pain. If pain persists, a local anesthetic injection is administered. After dermabrasion, hydrocolloid dressings (ABSOCURE® -SURGICAL, Nitto Denko Corporation, Osaka, Japan) are applied for one week to reduce postoperative pain and skin redness (Figure 1). No antibiotics or analgesics are required. The relationship between lidocaine tape application time and pain relief was investigated.

The study adhered to the principles of the Declaration of Helsinki. Ethical approval was obtained from the Review Board of the Japanese Society of Oral Care (approval no. E225002, April 10, 2025).

**Figure 2:**
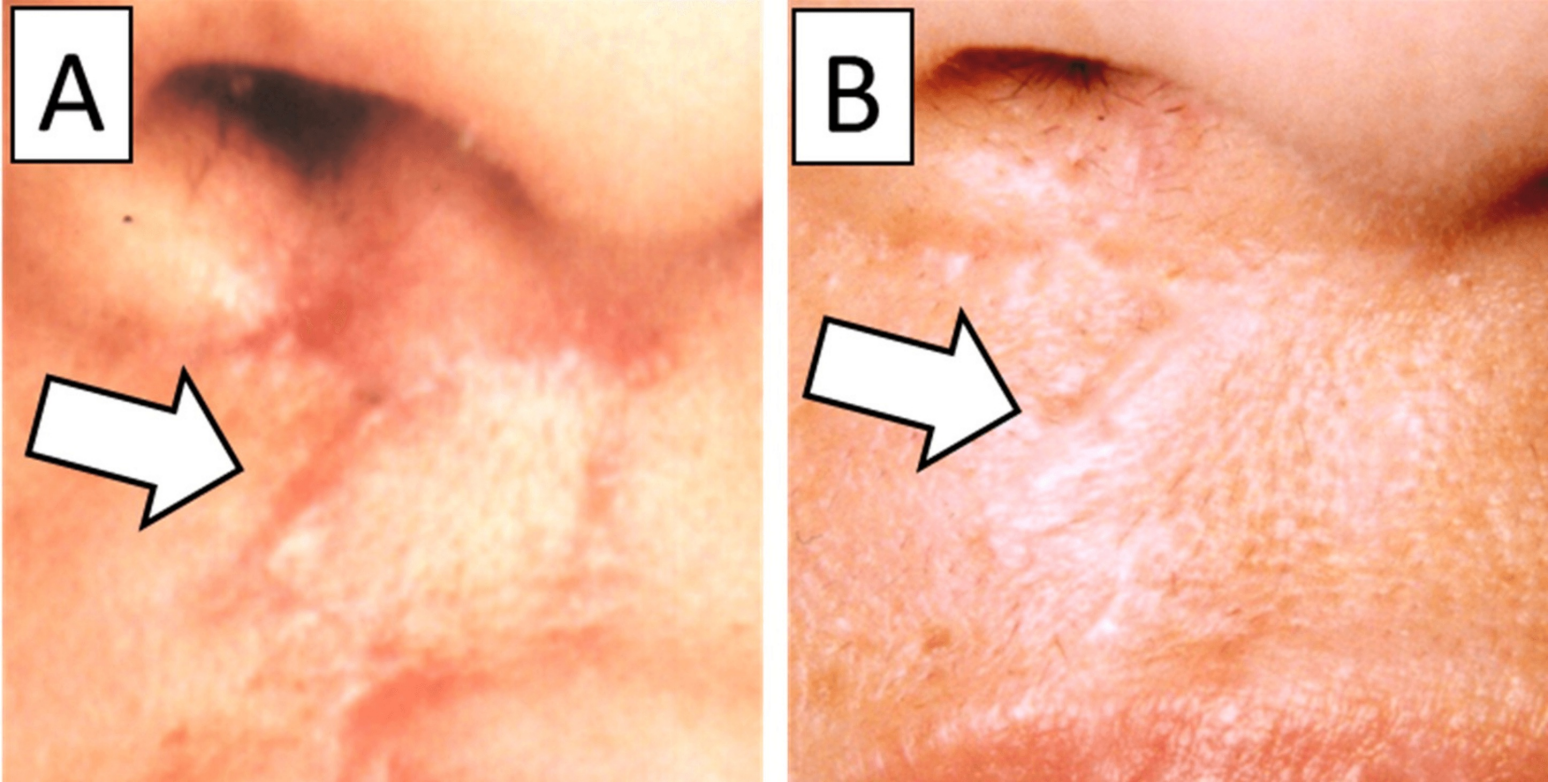
Effect of dermabrasion A: Preoperative condition of the scar. B: Post-dermabrasion condition of the scar. The deformity has improved.

Dermabrasion for post-cleft lip surgery under surface anesthesia was performed in 92 patients. All procedures were performed at the Cleft Lip and Palate Center, Aichi Gakuin University Dental Hospital. The procedure can be performed when the patient, not the guardian, expresses a desire for treatment and is old enough to cooperate with the surgery. It is carried out at least two years after the initial surgery, once the wound has stabilized and postoperative redness has subsided. There were no wound infections and complications, and good esthetic results were obtained in all patients (Figure 2).

In 16 cases where the lidocaine tape was applied for 30-59 minutes, painless treatment was possible in only four patients (25%), and in the remaining 12 patients, pain relief was obtained with the addition of lidocaine sponges. In 18 patients with a lidocaine tape application time of 60 to 89 minutes, painless treatment was possible in 11 patients (61%), and in seven cases, pain relief was obtained with the addition of a lidocaine sponge. In 58 patients with lidocaine tape applied for more than 90 minutes, painless treatment was possible in 39 patients (67%), and in 19 patients, pain relief was obtained with the addition of a lidocaine sponge. No patient required local anesthetic injection at any of the application times (Table 1).

**Table 1:** Pain-relieving effect of lidocaine tape

Application time	Penles® tape only	Addition of 4% lidocaine sponge	Addition of anesthetic injections	Total
30-50 minutes	4	12	0	16
60-89 minutes	11	7	0	18
>90 minutes	39	19	0	58
Total	54	38	0	92

## Discussion

Dermabrasion has long been utilized for the improvement of various cutaneous scars and irregularities, including those resulting from trauma, surgery, or acne [1-4]. While its utility is well-documented in general dermatologic and cosmetic contexts, its specific application in managing postoperative scarring after cleft lip surgery has not been thoroughly studied. This technical report contributes to this limited area of literature by evaluating the effectiveness and pain mitigation potential of surface anesthesia using lidocaine tape in pediatric and young adult patients.

Conventional dermabrasion is typically performed under injected local anesthesia, which, although effective, is associated with discomfort from needle insertion, a significant concern in children [7]. Topical anesthetic alternatives, such as refrigerant sprays or eutectic mixtures (e.g., EMLA cream [London, UK]), have been reported in the literature, but their onset time, efficacy, and potential for systemic absorption have raised concerns [8]. Penles® tape, a 60% lidocaine adhesive film, offers a needle-free alternative, but its use specifically for dermabrasion has not been systematically evaluated until now.

In our study, a clear correlation was observed between the application time of lidocaine tape and the degree of pain relief experienced during dermabrasion. Only 25% of patients with <60 minutes of application time reported painless treatment, compared to over 60% in those with ≥60 minutes of application. These findings suggest that the 30-minute guideline commonly cited for intradermal injections [7] may not be sufficient for procedures involving mechanical abrasion of the skin surface. This observation aligns with previous dermatologic data suggesting that deeper or prolonged procedures may require extended anesthetic contact time for adequate analgesia [6,7].

Comparative studies involving ablative laser resurfacing, a technique often considered an alternative to dermabrasion, have shown that although laser procedures may achieve similar or even superior cosmetic outcomes, they often necessitate more intensive anesthesia protocols and have higher equipment costs and training requirements [3,8]. In contrast, our approach using mechanical dermabrasion under surface anesthesia provides a low-cost, accessible, and well-tolerated option, particularly in resource-limited settings or among pediatric populations where procedural anxiety is high.

Furthermore, no patient in this study required injected local anesthesia, and there were no complications such as infection, prolonged erythema, or scarring beyond the intended resurfacing. This compares favorably with earlier dermabrasion literature, where complications such as prolonged erythema or hyperpigmentation have been reported at higher rates when deeper skin layers are inadvertently abraded [2,4].

In terms of postoperative management, the use of hydrocolloid dressings for one week post-procedure proved effective in minimizing discomfort and skin irritation. These dressings support wound healing by maintaining a moist environment and have been widely used in dermatologic and surgical applications [6]. Their integration into the dermabrasion protocol represents a practical and non-pharmacologic method to enhance recovery and patient satisfaction.

Recent studies supplement our findings and bolster the rationale for prolonged topical lidocaine application in minimally invasive procedures. A transmucosal lidocaine patch (46 mg/2 cm²) used for periodontal scaling showed significant pain reduction compared to placebo, with no adverse events, demonstrating that topical lidocaine can provide effective anesthesia during moderately invasive dermal procedures [9]. Meta-analyses of lidocaine/tetracaine patches used in various dermatologic settings (e.g., biopsies, minor resections) reveal significantly better pain relief compared to placebo when applied for 30-60 minutes, with minimal side effects [10]. These outcomes align with our results, which show that ≥60 minutes of lidocaine tape application led to painless dermabrasion in approximately 60% of patients, suggesting that longer contact times enhance analgesia beyond what is typically required for injections. Additional controlled trials in dental and mucosal contexts also indicate that lidocaine tape significantly reduces pain during needle puncture and infiltration anesthesia [11].

Cumulatively, this literature supports our recommendation of a 60-minute minimum application time for effective anesthesia in mechanical skin resurfacing, especially for areas with thicker or more innervated skin, like postoperative cleft lip scars.

Importantly, none of these studies reported serious systemic side effects. Our own experience corroborates this safety profile; there were no instances of lidocaine injection, infection, or delayed healing. Thus, our protocol, lidocaine tape applied for ≥60 minutes, supplemented with topical sponges as needed, offers a clinically sound and patient-friendly alternative. 

This report has some limitations. First, it requires a long time of 60 minutes before starting the procedure. Additionally, the differences between cases where pain relief is achieved quickly and those where complete analgesia is not obtained even after 60 minutes remain unclear. However, by demonstrating that this method is effective for children who refuse injections, clinically valuable insights have been gained.

## Conclusions

Dermabrasion under surface anesthesia using lidocaine tape is a safe and effective technique for managing postoperative scarring following cleft lip surgery. Our results indicate that applying lidocaine tape for at least 60 minutes significantly reduces procedural pain and eliminates the need for injectable anesthesia. No complications were observed, and all patients achieved favorable cosmetic outcomes. This minimally invasive approach offers a practical solution for improving patient comfort, particularly in pediatric populations, and may serve as a useful alternative to conventional anesthetic methods in scar management.
